# Quick and Simple Detection Technique to Assess the Binding of Antimicrotubule Agents to the Colchicine-Binding Site

**DOI:** 10.1007/s12575-010-9029-5

**Published:** 2010-04-08

**Authors:** Sébastien Fortin, Jacques Lacroix, Marie-France Côté, Emmanuel Moreau, Éric Petitclerc, René C-Gaudreault

**Affiliations:** 1 Unité des Biotechnologies et de Bioingénierie, Centre de recherche, C.H.U.Q., Hôpital Saint-François d'Assise, Québec, QC, G1L 3L5, Canada; 2 Faculté de Pharmacie, Université Laval, Pavillon Vandry, Québec, QC, G1V 0A6, Canada; 3 Université d'Auvergne, Inserm, U 990, F-63000 Clermont-Ferrand, France; 4 Faculté de Médecine, Université Laval, Pavillon Vandry, Québec, QC, G1V 0A6, Canada

**Keywords:** colchicine-binding site inhibitor, taxol-binding site inhibitor, *N*,*N*'-ethylene-bis(iodoacetamide), EBI, tubulin affinity assay, antimicrotubule agent

## Abstract

Development of antimitotic binding to the colchicine-binding site for the treatment of cancer is rapidly expanding. Numerous antimicrotubule agents are prepared every year, and the determination of their binding affinity to tubulin requires the use of purified tubulins and radiolabeled ligands. Such a procedure is costly and time-consuming and therefore is limited to the most promising candidates. Here, we report a quick and inexpensive method that requires only usual laboratory resources to assess the binding of antimicrotubules to colchicine-binding site. The method is based on the ability of *N*,*N*'-ethylene-bis(iodoacetamide) (EBI) to crosslink in living cells the cysteine residues at position 239 and 354 of β-tubulin, residues which are involved in the colchicine-binding site. The β-tubulin adduct formed by EBI is easily detectable by Western blot as a second immunoreacting band of β-tubulin that migrates faster than β-tubulin. The occupancy of colchicine-binding site by pertinent antimitotics inhibits the formation of the EBI: β-tubulin adduct, resulting in an assay that allows the screening of new molecules targeting this binding site.

## 1 Introduction

Antimicrotubule agents are important in the management of several cancers treatments notably breast, ovarian, and lung. Unfortunately, their effect is often hampered by chemoresistance, and they exhibit biopharmaceutical properties suitable for the treatment of only a limited number of cancers [1]. New antimicrotubule agents are therefore highly desirable, and several laboratories are eager to develop new drugs exhibiting improved antitumor efficacy and better biopharmaceutical properties [2]. One of the key end-points used in the screening of new molecules, beside the antiproliferative activity, is the binding affinity of the drug to one or another of the specific binding sites present on tubulin. Although binding sites for pironetin [3], tryprostatin [4], epothilone [5], *Vinca *[6], *Taxus *[7], and *Colchicum *[8] alkaloids have been identified so far, the colchicine-binding site remains the main target of numerous research programs. The determination and the characterization of the binding site(s) for each and every newly prepared drug to the latter binding sites are costly, time-consuming, and require specialized equipment, highly purified tubulin, and expensive radiolabeled ligands and are therefore applied only to the most promising molecules.

To circumvent these limitations, we have developed an inexpensive and simple detection technique to assess the binding of antimicrotubule agents acting on the colchicine-binding site. This method is based on the property of *N*,*N*'-ethylene-bis(iodoacetamide) (EBI), a homobifunctional thioalkylating agent, to crosslink the Cys-239 and the Cys-354 residues of β-tubulin involved in the colchicine-binding site [9]. The covalent binding of EBI to β-tubulin forms an adduct that is easily detected by Western blot as a second immunoreacting band of β-tubulin that migrates faster than the native β-tubulin band on SDS-PAGE (sodium dodecyl sulphate (SDS) polyacrylamide gel electrophoresis) [10]. Luduena has used that property to characterize, in vitro, the activity of several tubulin inhibitors [11,12]. In the course of our research program on 1-aryl-3-(2-chloroethyl)ureas [13], we found that the incubation of colchicine and iodoacetamide prevents the covalent binding of EBI in living cells resulting in the disappearance of the second immunoreacting band of β-tubulin on SDS-PAGE while vinblastine did not prevent the covalent binding of EBI. That observation provides us with a simple and inexpensive method to detect quickly and directly on living cells the ability of a molecule to bind to the colchicine-binding site without used of purified tubulin and the expensive [^3^H]-colchicine. Furthermore, the study being performed on living cells, it provides information on the ability of the drug to cross cell membranes, to diffuse through the cytosol and to react with dynamically functional microtubules that are in contact with several cytoskeleton-associated proteins, information that are unavailable when using only purified tubulins in a in vitro setting.

In this study, we assessed the robustness of the method using: (1) drugs binding to the three main binding sites found on the α-,β-tubulin heterodimer, namely the taxol-, the colchicine-, and the vinca-binding sites, (2) drugs unrelated to tubulins and the cytoskeleton such as daunorubicin and verapamil, and (3) four cell lines found in most laboratories. As shown in Table 1, in Additional file 1, drugs binding to the colchicine-binding site inhibit the bisthioalkylation of β-tubulin by EBI and consequently the formation of the EBI: β-tubulin adduct and the second immunoreacting β-tubulin band. Conversely, vinca-binding site inhibitors and tubulin-unrelated drugs did not inhibit the covalent binding of EBI and the formation of the second immunoreacting β-tubulin band. Of note, the distinct colchicine-binding site and the paclitaxel-binding site are close (16–17 Å) and share a slight overlap between their interaction domains [14]. This overlap between the colchicine-binding site and the paclitaxel-binding site seems to explain the effect of paclitaxel when challenged by EBI, as seen in Table 1, 1 and may show a screening advantage for dual inhibitors. In addition, as shown in Table 2, in Additional file 2, the assay can be performed not only with MDA-MB-231 but also with cell lines such as HT-29, M21, and MCF-7 that contain β-tubulin isoforms bearing the Cys-239 and the Cys-354 residues.

**Figure 1:**
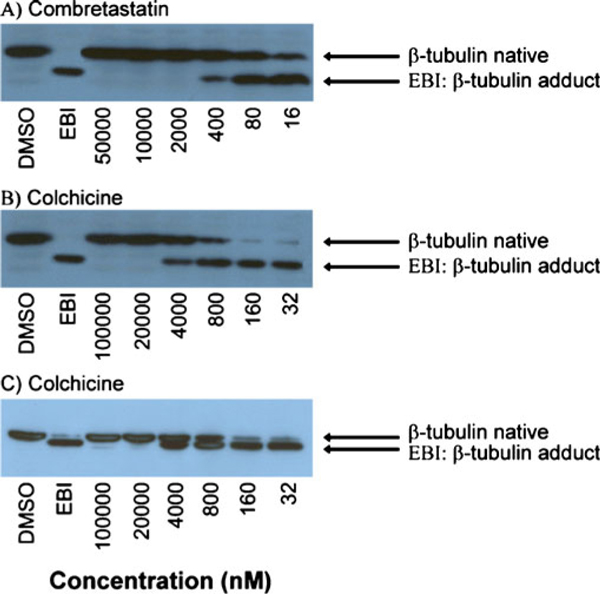
**Effect of escalating concentrations of combretastatin A-4 and colchicine on the inhibition of the bisthioalkylation of Cys-239 and Cys-354 of β-tubulin by *N*,*N*'-ethylene-bis(iodoacetamide) on MDA-MB-231 cell lines**. The experiments for panels **A** and **B** were performed using SDS 65% while SDS 98.5% was used for panel **C**.

As illustrated in Figure 1, the inhibition of EBI binding to tubulin is concentration-dependant. When the experiments are performed using escalating concentrations of colchicine and combretastatin A-4 (10 to 31 250-fold the IC_50_), a semi-quantitative assessment of the affinity of the drug for the binding site is possible. Moreover, the purity of the sodium dodecyl sulfate (SDS) used to perform the experiments is crucial, since SDS at 65% significantly improved the separation between native β-tubulin and the EBI: β-tubulin adduct.

## 2 Materials and Equipments

Human breast carcinoma MDA-MB-231, human colon carcinoma HT-29, human skin melanoma M21, and human breast carcinoma MCF-7 cells were obtained from the American Type Culture Collection (ATCC HTB-26; Manassas, VA). Cells were grown in high glucose Dulbecco's Modified Eagle Medium supplemented with 5% (*v*/*v*) defined bovine calf serum (Hyclone laboratories, Logan, UT). Cells were maintained in a moisture-saturated atmosphere at 37°C in 5% of CO_2_. Biochemicals and the monoclonal anti-β-tubulin antibody (clone TUB 2.1) were purchased from Sigma-Aldrich (St. Louis, MO) and used as received unless specified otherwise. SDS at 98.5% and SDS at 65% of purity were purchased from Sigma-Aldrich. The enhanced chemiluminescence Western blotting detection reagent kit was provided by Amersham Canada (Oakville, Canada). Mixtures are expressed as volume/volume ratios unless indicated otherwise. Daunorubicin was purchased from Rhône-Poulenc (Courbevoie, France) and combretastatin A-4 was synthesized according to Petit et al. [15]. EBI was obtained from TRC Biomedical Research Chemicals (North York, ON, Canada). All drugs were dissolved in dimethylsulfoxide, and the final concentration of dimethylsulfoxide in the culture medium was maintained under 0.5%.

## 3 Methods

### 3.1 Cell Culture and Treatment

Prior to drug exposition, 6-well plates were seeded with either MDA-MB-231, HT-29, M21, or MCF-7 cells at 7 × 10^5^ cells per well and incubated for 24 h. Cells were first incubated in presence of approximately 1,000-times the IC_50_ of the drugs for 2 h and afterward treated with EBI (100 μM, final concentration) for 1.5 h at 37°C without changing the culture medium, which contains the drugs. The control cells were treated with 0.5% dimethylsulfoxide. Afterward, floating and adherent cells were harvested using a rubber policeman and centrifuged for 3 min at 8,000 rpm. The pellets were washed with 500 μL of cold phosphate-buffered saline and stored at -80°C until use.

### 3.2 Western Blot

The cells pellets were lysed by addition of 100 μL of Laemmli sample buffer 5× (60 mM Tris–Cl pH 6.8, 2% SDS, 10% glycerol, 5% β-mercaptoethanol, 0.01% bromophenol blue). Cell extracts were boiled for 5 min. The protein concentration was determined using the Bradford method. Twenty micrograms of proteins from the protein extracts were subjected to electrophoresis using 0.1% SDS (98.5% or 65% of purity) and 10% polyacrylamide gels. The proteins were transferred onto nitrocellulose membranes that were incubated with TBSMT (Tris-buffered saline + 0.1% (*v*/*v*) Tween-20 with 2.5% fat-free dry milk) for 1 h at room temperature and then with the anti-β-tubulin primary antibody in TBSMT (1:500) for 16 h at 4°C. Membranes were washed with TBST (Tris-buffered saline with + 0.1% (*v*/*v*) Tween-20) and incubated with peroxidase-conjugated anti-mouse immunoglobulin in TBSMT (1:2,500) for 2.5 h at room temperature. After washing the membranes with TBST, detection of the immunoblot was carried out with an enhanced chemiluminescence detection reagent kit.

## 4 Summarized Stepwise Protocol

1. Seeding 6-well plates with either MDA-MB-231, HT-29, M21, or MCF-7 cells at 7 × 10^5^ cells per well and incubation for 24 h

2. Incubation of the cells for 2 h with the drug to be tested and then with 100 μM of EBI for 1.5 h

3. Harvesting, pooling, and freezing of both floating and adherent cells

4. Lysis of cells with Laemmli sample buffer and boiling the samples for 5 min

5. Separation of the protein extracts by electrophoresis using 10% polyacrylamide gels (100 V, 2.5 h)

6. Transfer of the proteins onto nitrocellulose membrane (35 V, 16 h at 4°C)

7. Incubation of the nitrocellulose membrane with TBSMT for 1 h

8. Incubation of the nitrocellulose membrane with the primary antibody anti-β-tubulin for 16 h at 4°C then washing five times with TBST

9. Incubation of the nitrocellulose membrane with the peroxidase-conjugated anti-mouse immunoglobulin for 2.5 h at room temperature and then washing five times with TBST

10. Detection of β-tubulin bands using an enhanced chemiluminescence detection reagent kit

## 5 Discussion of Key Steps in the Protocol

Concentration and duration of the incubation of the tested drug with the cells may change from one class of compound to another. However, all drugs tested so far have produced good signals when using the aforementioned experimental conditions. The purity of the SDS used for the electrophoresis seems critical for optimal separation of the β-tubulin and the EBI: β-tubulin adduct. In our hand, the SDS at 65% of purity supplied by Sigma-Aldrich was optimal (Figure 1).

## Supplementary Material

Additional file 1 Click here for file

Additional file 2 Click here for file
