# Supplementary figures and images for: Quick and Simple Detection Technique to Assess the Binding of Antimicrotubule Agents to the Colchicine-Binding Site
Source: Biol Proced Online. 2010 Apr 8;12:113–7. doi: 10.1007/s12575-010-9029-5 (PMC3055821; doi:10.1007/s12575-010-9029-5)

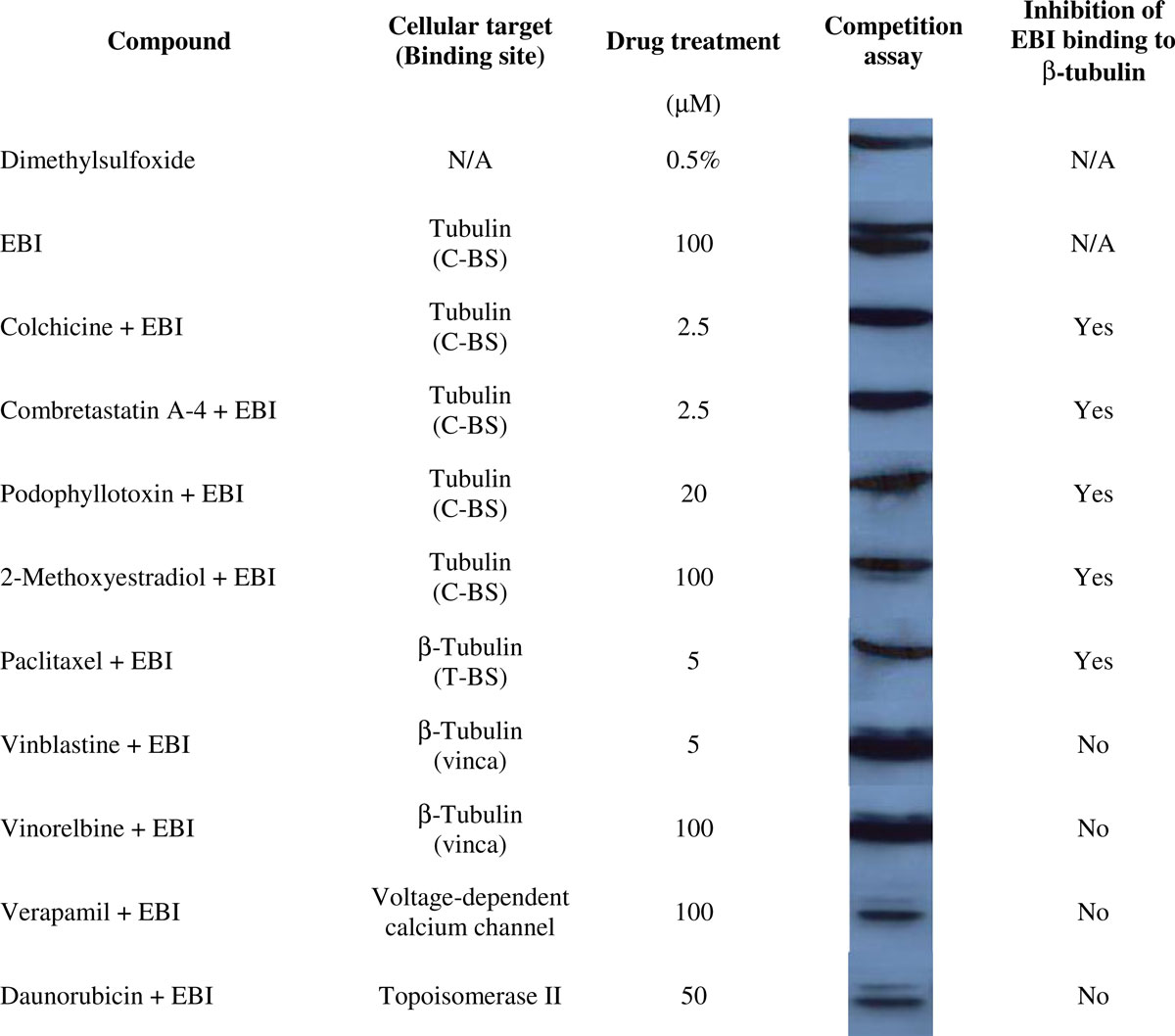

Supplement: Additional file 1 [file 1480-9222-12-1-9029-S1.jpg]

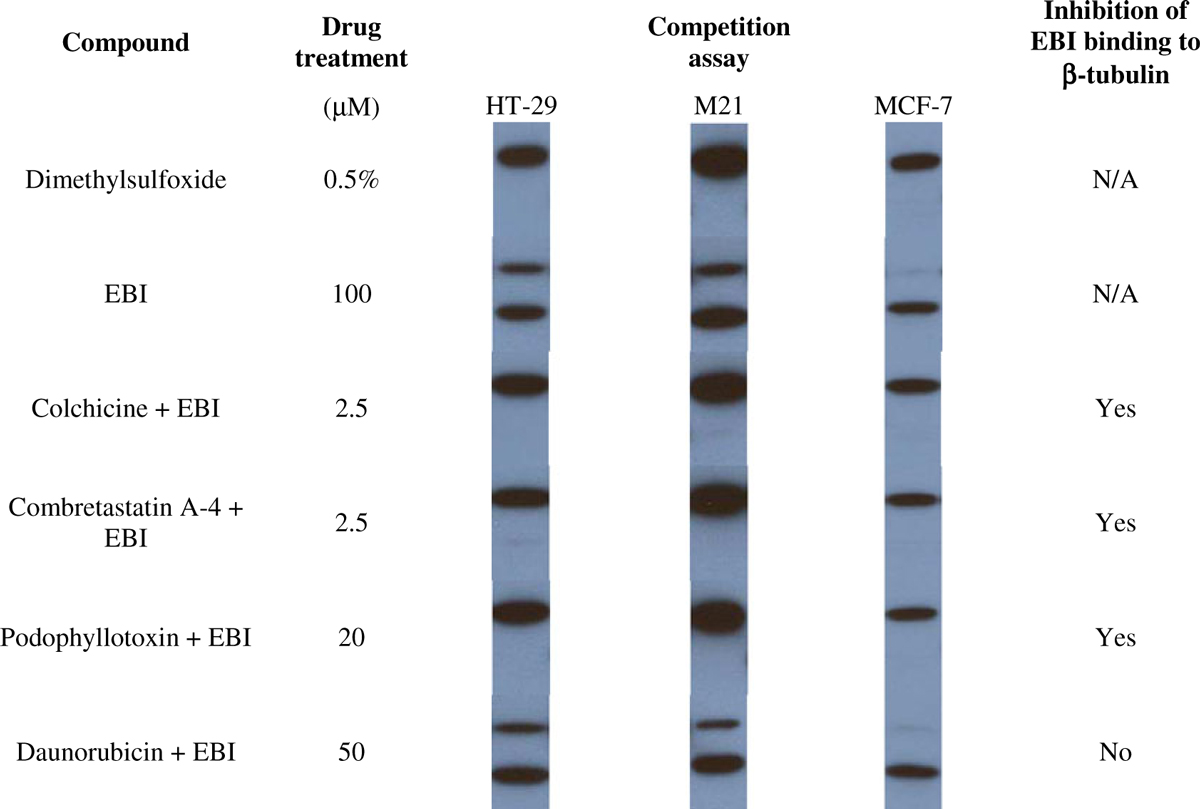

Supplement: Additional file 2 [file 1480-9222-12-1-9029-S2.jpg]
